# Two-dimensional TaS_2_ as a contact material for MXene Sc_2_CF_2_ semiconductors: a first-principles study

**DOI:** 10.1039/d5ra05385d

**Published:** 2025-10-20

**Authors:** Tuan V. Vu, Phan T. T. Huyen, Nguyen N. Hieu, Huynh V. Phuc, Chuong V. Nguyen

**Affiliations:** a Laboratory for Computational Physics, Institute for Computational Science and Artificial Intelligence, Van Lang University Ho Chi Minh City Vietnam tuan.vu@vlu.edu.vn; b Faculty of Mechanical, Electrical, and Computer Engineering, Van Lang School of Technology, Van Lang University Ho Chi Minh City Vietnam; c Hue FPT School Hue Vietnam; d Institute of Research and Development, Duy Tan University Da Nang 550000 Vietnam hieunn@duytan.edu.vn; e Faculty of Natural Sciences, Duy Tan University Da Nang 550000 Vietnam; f Division of Physics, School of Education, Dong Thap University Dong Thap 870000 Vietnam hvphuc@dthu.edu.vn; g Department of Materials Science and Engineering, Le Quy Don Technical University Hanoi 100000 Vietnam

## Abstract

Metal–semiconductor heterojunctions are fundamental to modern electronics, serving as the key interface for charge transport and enabling diverse functionalities in electronic and optoelectronic devices. In this work, we computationally design the electrical contact architecture by vertically integrating two-dimensional TaS_2_ and Sc_2_CF_2_ materials using first-principles predictions. The TaS_2_/Sc_2_CF_2_ heterostructure is predicted to be energetically and thermally stable at room temperature and characterized by weak van der Waals interactions. Additionally, the integration of TaS_2_ with Sc_2_CF_2_ enhances the mechanical rigidity of the heterostructure. More interestingly, the TaS_2_/Sc_2_CF_2_ heterostructure forms a Schottky contact with an electron barrier of 0.36 eV. Furthermore, it exhibits remarkable tunability in electronic properties and contact behavior under an applied electric field. Specifically, the electric field induces a transition from Schottky to ohmic contact, as well as a conversion from n-type to p-type Schottky contact. This tunability signifies a barrier-free charge injection process, making the TaS_2_/Sc_2_CF_2_ heterostructure a promising candidate for next-generation electronic and optoelectronic devices.

## Abstract 2

Metal–semiconductor heterojunctions are fundamental to modern electronics, serving as the key interface for charge transport and enabling diverse functionalities in electronic and optoelectronic devices.

## Graphical abstract

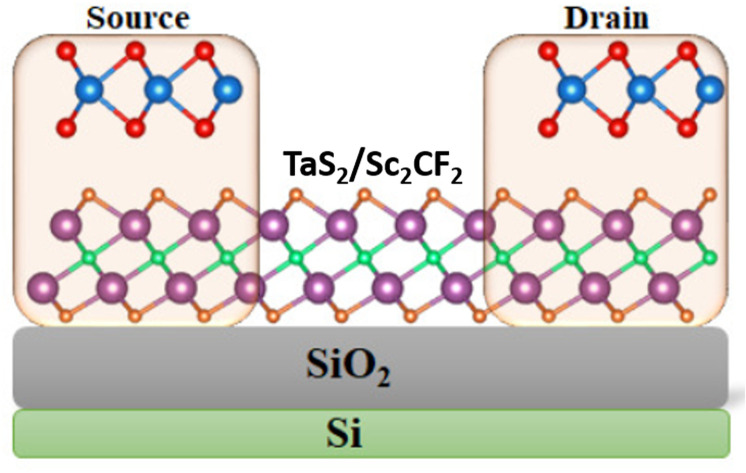

## 1 Introduction

Two-dimensional (2D) materials have revolutionized the field of electronic devices due to their unique structures and extraordinary properties.^1,2^ Since the discovery of graphene,^3^ extensive research has led to the exploration of various 2D material families, each offering distinct advantages. Hexagonal boron nitride (*h*-BN), a wide-bandgap insulator,^4^ serves as an ideal dielectric substrate due to its atomically smooth surface and minimal charge trapping effects.^5,6^ Transition metal dichalcogenides (TMDCs),^7^ with their tunable band gaps^8,9^ and high carrier mobility,^10^ have enabled advancements in optoelectronic devices^11^ and low-power transistors.^12,13^ Meanwhile, MXenes,^14^ a versatile class of layered transition metal carbides and nitrides, exhibit mechanically robust,^15,16^ broadening their potential for energy storage and catalysis.^17,18^ These diverse functionalities pave the way for innovative heterostructures, where synergistic interactions between 2D materials can be harnessed to tailor electronic and interfacial properties for next-generation applications.^19,20^

Among the diverse family of TMDCs, tantalum disulfide (TaS_2_) stands out due to its intrinsic metallic nature and intriguing electronic properties.^21^ Unlike semiconducting TMDCs, TaS_2_ exhibits a highly conductive behavior attributed to its layered structure and strong electron correlation effects.^22^ Its metallic state enables efficient charge transport, making it a promising candidate for applications in interconnects, low-resistance contacts, and electronic devices requiring high conductivity.^23^ Moreover, the integration of metallic TaS_2_ with semiconducting 2D materials opens new avenues for designing high-performance heterostructures with tailored electronic and interfacial properties.^24–26^

Similar to metallic TaS_2_, most MXenes exhibit intrinsic metallic behavior.^27^ However, Sc_2_CF_2_ stand out as rare exceptions, displaying semiconducting characteristics due to their unique surface terminations and structural modifications.^28^ Owing to these intriguing properties, Janus MXenes have recently been predicted to be promising candidates for applications in nanoelectronics, optoelectronics, and energy conversion devices, further broadening the potential of Sc-based MXenes.^29–31^ In particular, Sc_2_CF_2_, with its well-defined band gap^32^ and tunbale electronic properties,^33,34^ emerges as an effective semiconducting channel for integration with other 2D materials.^29,35–38^ Therefore, in this work, we employ first-principles calculations to design and analyze the electrical contact between metallic TaS_2_ and semiconducting Sc_2_CF_2_, aiming to establish a high-performance metal–semiconductor heterostructure with tailored electronic properties. Our results reveal that the TaS_2_/Sc_2_CF_2_ heterostructure naturally forms a p-type Schottky contact with a relatively low barrier height of 0.36 eV. More importantly, this contact behavior can be effectively tuned by an external electric field, which induces a transition between n-type and p-type Schottky contacts. It should be noted that the Schottky junction plays a vital role in modern electronics and spintronics owing to its ability to control carrier injection and band alignment at metal–semiconductor interfaces, which directly determines the performance of transistors, photodetectors, and nanoelectronic devices. These results provide valuable insights into the interfacial physics of the TaS_2_/Sc_2_CF_2_ heterostructure, paving the way for the design of next-generation electronic devices, such as field-effect transistors and photodetectors with enhanced efficiency and functionality based on the TaS_2_/Sc_2_CF_2_ heterostructure.

## 2 Computational methods

To investigate the electronic properties and contact characteristics of the TaS_2_/Sc_2_CF_2_ heterostructure, we performed first-principles calculations based on density functional theory (DFT) as implemented in the Vienna *Ab initio* Simulation Package (VASP).^39^ The exchange–correlation interactions were treated using the generalized gradient approximation (GGA) in the Perdew–Burke–Ernzerhof (PBE) functional.^40^ Additionally, van der Waals (vdW) interactions were accounted for by employing the DFT-D3 correction method^41^ to ensure accurate representation of interlayer interactions. The plane-wave basis set was used with a kinetic energy cutoff of 510 eV to ensure sufficient convergence in electronic structure calculations. A 12 × 12 × 1 Monkhorst–Pack *k*-point mesh^42^ was employed for Brillouin zone sampling. To model the TaS_2_/Sc_2_CF_2_ heterostructure, a vacuum layer of 25 Å was introduced to eliminate spurious interactions between periodic images. The atomic structures were fully optimized until the total energy was converged to within 10^−8^ eV and the residual force on each atom was smaller than 0.01 eV Å^−1^. To evaluate the structural stability of the TaS_2_/Sc_2_CF_2_ heterostructure, both *ab initio* molecular dynamics (AIMD)^43^ simulations and phonon dispersion calculations were carried out. The AIMD simulations were performed within the canonical (NVT)^44^ ensemble using a Nosé–Hoover thermostat at 300 K for a total simulation time of 9 ps with a time step of 1 fs. In addition, the phonon spectra were calculated using density functional perturbation theory (DFPT).^45^

## 3 Results and discussion

**Fig. 1:**
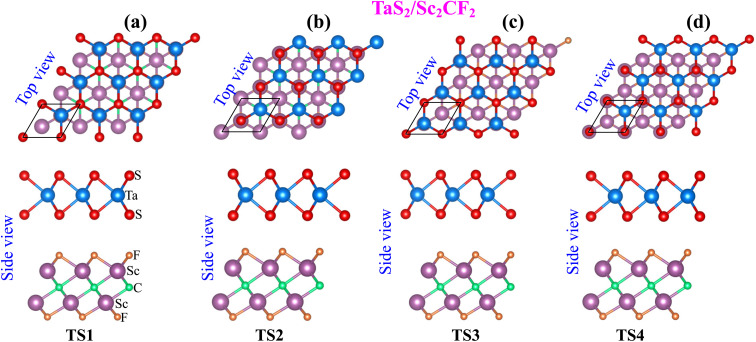
Optimized atomic structures of the TaS_2_/Sc_2_CF_2_ heterostructure for different stacking arrangements of (a) TS1, (b) TS2, (c) TS3 and (d) TS4. Red and blue balls represent the S and Ta atoms, respectively. While, orange, violet and green balls stand for the F, Sc and C atoms, respectively.

The TaS_2_/Sc_2_CF_2_ heterostructure is designed *via* vertical integration of a (1 × 1) unit cell of TaS_2_ and a (1 × 1) unit cell of Sc_2_CF_2_ monolayer. This design is facilitated by their minimal lattice mismatch, with TaS_2_ exhibiting a lattice constant of 3.31 Å and Sc_2_CF_2_ having a slightly larger value of 3.34 Å. The atomic structures of the TaS_2_/Sc_2_CF_2_ heterostructure are illustrated in Fig. 1. Due to the hexagonal symmetry of the TaS_2_ and Sc_2_CF_2_ monolayers, many of these possible arrangements are symmetry-equivalent or can be obtained by simple in-plane translations of the constituent monolayers. Therefore, we focused on four representative four stacking arrangements, which capture the physically distinct registries, namely on-top, hollow, and bridge-like configurations, as illustrated in Fig. 1. The interlayer spacing *d* between the lowest sulfur (S) layer and the topmost fluorine (F) layer has been calculated. Among the configurations, the TS2 stacking exhibits the shortest interlayer spacing at 2.72 Å, while the TS1 stacking shows the largest spacing of 3.29 Å. It is evident that the obtained interlayer spacing *d* remains larger than the sum of the covalent bond lengths of fluorine (0.57 Å) and sulfur (1.05 Å) atoms. This observation suggests that the TaS_2_/Sc_2_CF_2_ heterostructure is primarily stabilized by weak van der Waals (vdW) interactions rather than strong chemical bonding. Furthermore, these values of *d* are also comparable with those obtained in other vdW typical heterostructures,^35,37,38,46^ further confirming the weak vdW interactions in the TaS_2_/Sc_2_CF_2_ heterostructure. Furthermore, the binding energy is also evaluated to quantify the stability of the TaS_2_/Sc_2_CF_2_ heterostructure. The binding energy per unit cell (*E*_b_) is calculated using the formula:1
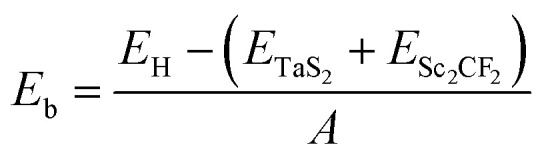
where *E*_H_ represents the total energy of the TaS_2_/Sc_2_CF_2_ system, *E*_TaS_2__ and *E*_Sc_2_CF_2__ correspond to the individual total energies of the monolayers, and *A* denotes the area of the heterostructure. A negative binding energy indicates an energetically favorable interaction, confirming the structural stability driven by vdW forces. Among the stacking configurations, TS_2_ exhibits the lowest binding energy of Eb = −18.73 meV Å−2, indicating the most stable arrangement. In contrast, the TS_1_ stacking presents the highest binding energy of Eb = −11.45 meV Å−2. These results highlight the TS_2_ stacking as the most energetically favorable configuration for the TaS_2_/Sc_2_CF_2_ heterostructure. Additionally, the electron localization function (ELF) for the TS2 stacking configuration is visualized to describe the bonding nature of the TaS_2_/Sc_2_CF_2_ heterostructure, as depicted in Fig. S1 of the SI. It is obvious that the high ELF values (close to 1.0) are mainly localized around the S and F atoms, indicating strong covalent bonding within the TaS_2_ and Sc_2_CF_2_ layers. In contrast, the interfacial region between TaS_2_ and Sc_2_CF_2_ layers exhibits very low ELF values (close to 0.0), confirming that the interaction across the interface is weak and dominated by vdW forces. To gain deeper insights into the stability and electronic behavior of the TS_2_ stacking, we extend our analysis to its mechanical properties, band structures, and charge redistribution.

**Fig. 2:**
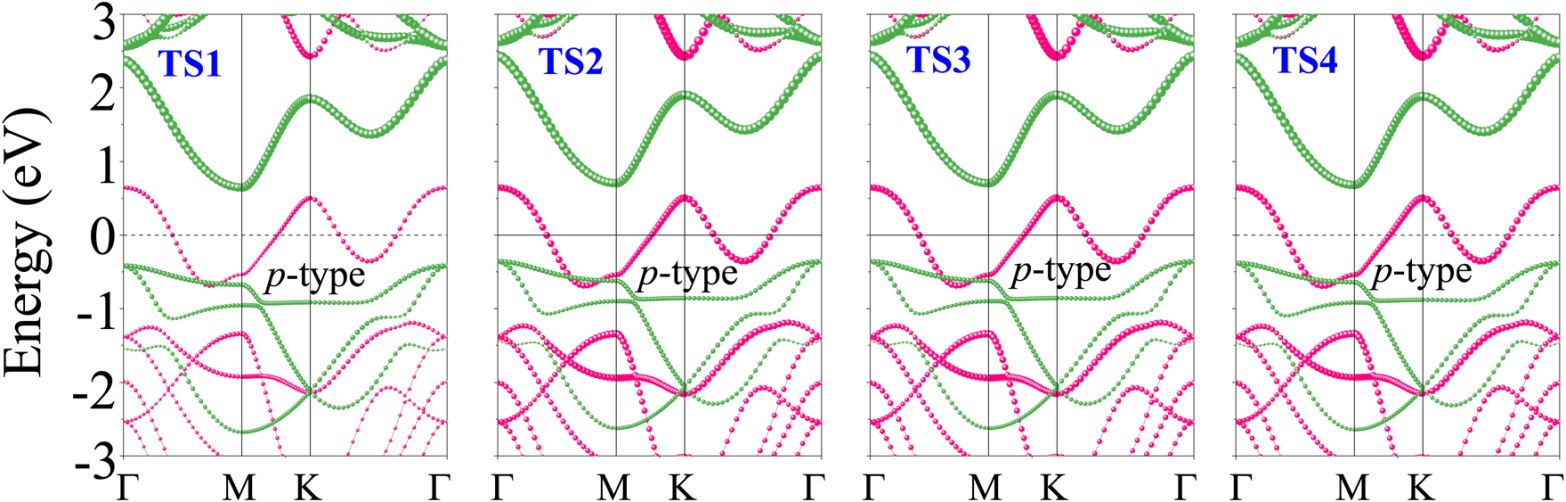
Projected band structures of the TaS_2_/Sc_2_CF_2_ heterostructure. Purple and green lines represent the projections of the band structures of TaS_2_ and Sc_2_CF_2_ layers, respectively. The Fermi level is set to be zero.

The projected band structures of the TaS_2_/Sc_2_CF_2_ heterostructure are illustrated in Fig. 2. It is evident that the TaS_2_/Sc_2_CF_2_ heterostructure exhibits metallic behavior, characterized by crossing bands at the Fermi level. Additionally, the band structures of the TaS_2_/Sc_2_CF_2_ heterostructure, irrespective of the stacking arrangement, closely resemble the sum of those of the individual monolayers, as illustrated in Fig. S2. Besides, it is evident that the band crossing at the Fermi level of the heterostructure is primarily contributed by the Ta atoms from the TaS_2_ layer. On the other hand, the CB of the Sc_2_CF_2_ layer originates mainly from the Sc atom, while its VB is due to the orbital hybridization between C and F atoms, as illustrated in Fig. S3. This band alignment mechanism arises due to the weak vdW interactions between the TaS_2_ and Sc_2_CF_2_ layers, preserving the electronic characteristics of the constituent materials. Furthermore, the metal–semiconductor TaS_2_/Sc_2_CF_2_ heterostructure is primarily characterized by the formation of either Schottky or ohmic contact. As illustrated in Fig. 2, the metallic TaS_2_ layer exhibits band crossings at the Fermi level, while the Fermi level is positioned between the conduction and valence band edges of the Sc_2_CF_2_ layer. This band alignment suggests the formation of a Schottky contact at the TaS_2_/Sc_2_CF_2_ heterostructure. The Schottky barriers of the TaS_2_/Sc_2_CF_2_ heterostructure are defined as follows:

2 *Φ*_e_ = *E*_c_ − *E*_F_, *Φ*_h_ = *E*_F_ − *E*_v_

where *Φ*_e_ and *Φ*_h_ represent the Schottky barrier heights for electrons and holes, respectively. *E*_c_ and *E*_v_ denote the conduction and valence band edges of the Sc_2_CF_2_ semiconductor, while *E*_F_ is the Fermi level of the heterostructure. The calculated barrier heights provide insights into charge carrier transport mechanisms at the interface. Our results reveal that the TS2 configuration has the narrowest *Φ*_h_ of 0.36 eV and highest *Φ*_e_ of 0.71 eV. While the TS1 configuration has the highest *Φ*_h_ of 0.41 eV and narrowest *Φ*_e_ of 0.65 eV. A lower Schottky barrier facilitates electron injection and enhances conductivity, whereas a higher barrier height restricts charge flow, influencing the electronic properties of the heterostructure. These values of the Schottky barriers are comparable with those in other 2D vdW metal–semiconductor heterostructures.^47,48^ In the TaS_2_/Sc_2_CF_2_ heterostructure, the barriers for holes *Φ*_h_ is consistently lower than that for electrons (*Φ*_e_), indicating the formation of an p-type Schottky contact. Furthermore, to validate the accuracy of the calculated method, we provide the HSE06 projected band structure of the TaS_2_/Sc_2_CF_2_ heterostruture for the TS2 stacking configuration, as illustrated in Fig. S3 of the SI. The *Φ*_h_ and *Φ*_e_ are obtained to be 0.38 and 1.48 eV, respectively, confirming the formation of the p-type Schottky contact. In addition, since the difference between the HSE06 and PBE results is relatively small, we have opted to employ the PBE method for the remaining calculations to ensure computational efficiency. This type of contact is advantageous for efficient hole transport across the interface, making the heterostructure highly promising for electronic and optoelectronic applications, such as field-effect transistors (FETs) and photodetectors.

**Fig. 3:**
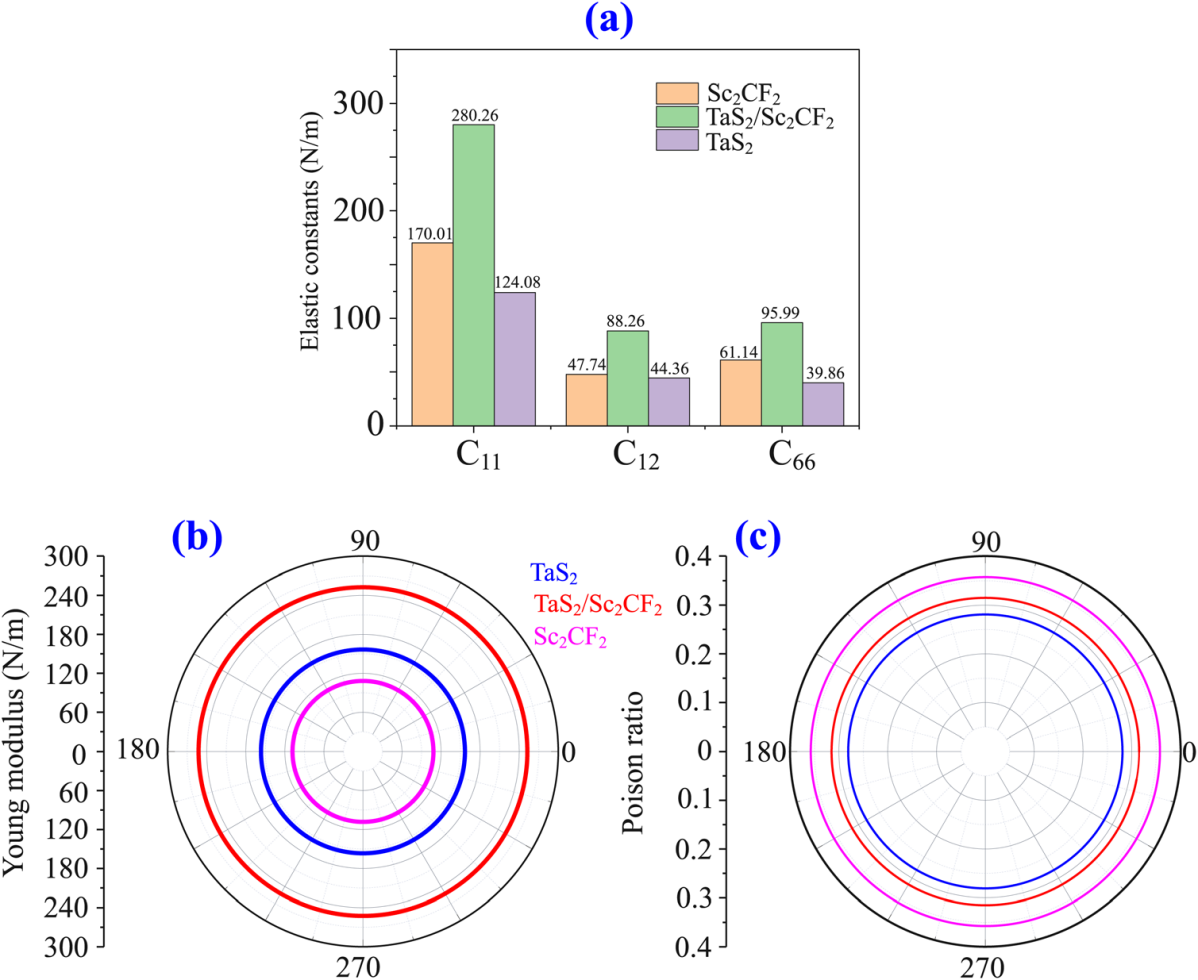
(a) Calculated elastic constants (b) Young modulus and (c) Poison ratio of the TaS_2_/Sc_2_CF_2_ heterostructure and the constituent monolayers.

In order to examine the mechanical stability, we performed elastic constant calculations using the energy-strain technique through the harmonic approximation, expressed as:^49^3
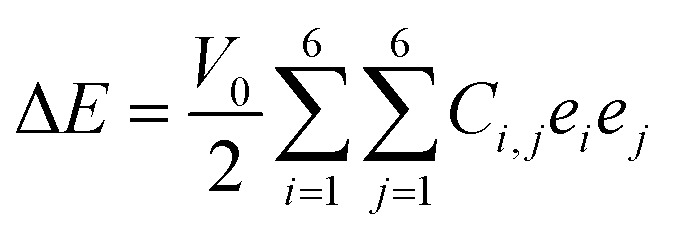
where Δ*E* and *V*_0_ represent the energy and volume differences between the strained and unstrained unit cells, respectively, while the strain vector components are denoted as *e*_*i*_ and *e*_*j*_. The TaS_2_/Sc_2_CF_2_ heterostructure possesses four independent elastic constants: *C*_11_, *C*_22_, *C*_12_, and *C*_66_. Specifically, *C*_11_ and *C*_22_ denote in-plane stiffness against uniaxial strain along the *x* and *y* direction, respectively, while *C*_12_ describes the coupling between strain along the *x* direction and the induced stress along the *y* direction. It determines how deformation in the *x* direction induces stress in the *y* direction. Because of the hexagonal symmetry, *C*_11_ = *C*_22_, which ensures that the in-plane Young's modulus and Poison ration are isotropic. The shear constant *C*_66_ characterizes the resistance to in-plane shear deformation and, for hexagonal symmetry, is not independent but can be expressed as *C*_66_ = (*C*_11_ − *C*_12_)/2. These values, along with those of the TaS_2_ and Sc_2_CF_2_ monolayers, are presented in Fig. 3(a). Notably, the TaS_2_/Sc_2_CF_2_ heterostructure exhibits larger elastic constants *C*_*ij*_ compared to its constituent monolayers, indicating enhanced mechanical rigidity. Furthermore, the obtained elastic constants satisfy the Born-Huang stability criteria (*C*_11_ > *C*_12_ and *C*_66_ > 0), confirming the mechanical stability of the heterostructure. This improvement enables the material to better withstand external mechanical stress and deformation while maintaining its structural integrity. To further assess the mechanical properties of the TaS_2_/Sc_2_CF_2_ heterostructure, we evaluate Young's modulus (*Y*) and Poisson's ratio (*ν*), which provide insights into the material's stiffness and deformation characteristics.^50^4
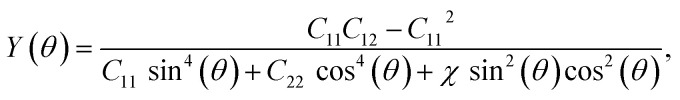
5
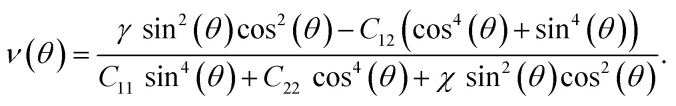
where 
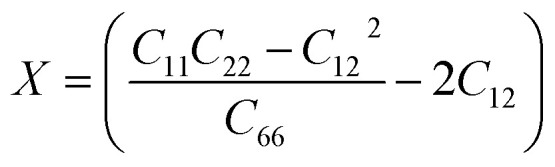
 and 
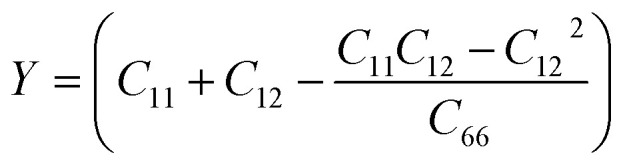
. *γ* represents the in-plane orientation angle of the applied strain. The calculated values of Young's modulus and Poisson's ratio for the TaS_2_/Sc_2_CF_2_ heterostructure, along with those for the individual TaS_2_ and Sc_2_CF_2_ monolayers, are presented in Fig. 3(b and c). Notably, the heterostructure exhibits an enhanced Young's modulus compared to its constituent monolayers, indicating improved mechanical strength. The Young's modulus of the TaS_2_/Sc_2_CF_2_ heterostructure is calculated to be 252.46 N m^−1^, which is comparable to that of other van der Waals (vdW) heterostructures.^51–53^ Meanwhile, its Poisson's ratio is obtained to be 0.31, remaining within a favorable range (*ν* < 0.5), ensuring structural integrity under applied stress. Furthermore, since this value is larger than the conventional ductile–brittle threshold of 0.26,^54^ the TaS_2_/Sc_2_CF_2_ heterostructure can be classified as ductile. These characteristics make the TaS_2_/Sc_2_CF_2_ heterostructure a promising candidate for applications requiring robust and flexible 2D materials.

**Fig. 4:**
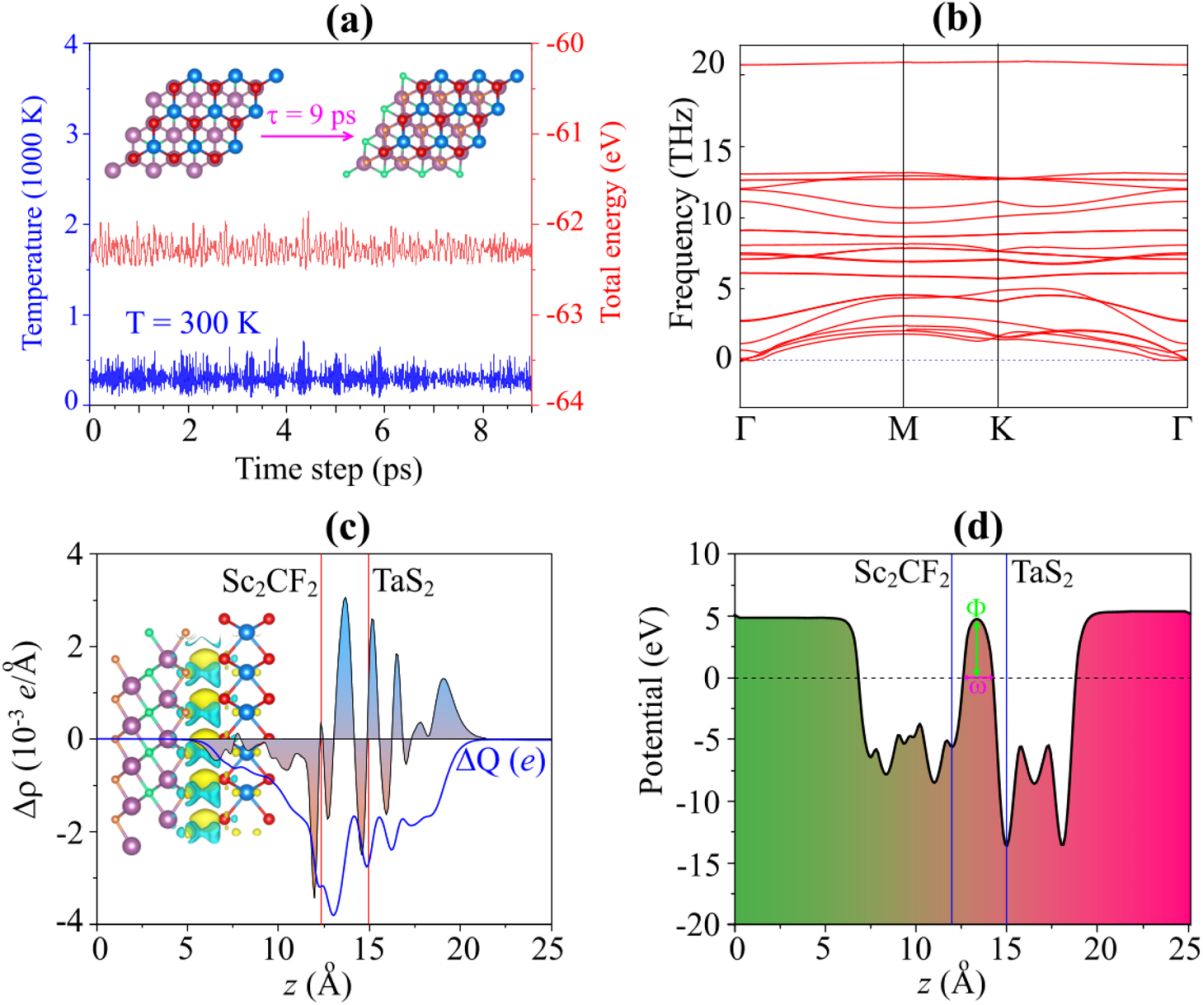
(a) Temperature and total energy variations of the TaS_2_/Sc_2_CF_2_ heterostructure obtained from AIMD simulations, (b) phonon spectra (c) planar-averaged charge density distribution and (d) electrostatic potential profile of the TaS_2_/Sc_2_CF_2_ heterostructure. Charge accumulation and depletion regions are visualized in yellow and cyan, respectively.

To assess the thermal stability of the TaS_2_/Sc_2_CF_2_ heterostructure, we conducted *ab initio* molecular dynamics (AIMD) simulations and phonon spectra, as depicted in Fig. 4(a and b). The temperature and total energy fluctuations were monitored throughout the simulation period to evaluate the robustness of the structure under thermal perturbations. The absence of significant structural distortions or energy divergence confirms the thermal stability of the heterostructure, indicating its potential viability for practical applications. In addition, the variations in total energy and temperature remain relatively small throughout the simulation, further affirming the structural integrity of the TaS_2_/Sc_2_CF_2_ heterostructure under thermal perturbations, confirming the thermal stability. It is obvious from Fig. 4(b) that the absence of imaginary phonon modes throughout the Brillouin zone confirms the dynamical stability of the heterostructure. All these findings confirm the thermal and dynamical stability of the TaS_2_/Sc_2_CF_2_ heterostructure at room temperature.

To examine charge redistribution at the TaS_2_/Sc_2_CF_2_ interface, we calculate the planar-averaged charge density difference, as shown in Fig. 4(b). The charge density difference (Δ*ρ*) is obtained by subtracting the charge densities of the isolated TaS_2_ and Sc_2_CF_2_ monolayers from that of the combined heterostructure (*ρ*_H_):

6 Δ*ρ* = *ρ*_H_ − (*ρ*_TaS_2__ + *ρ*_Sc_2_CF_2__).

One can observe a notable charge redistribution near the contact region, indicating the presence of interlayer interactions facilitated by van der Waals (vdW) forces. Furthermore, positive charge accumulation occurs near the Sc_2_CF_2_ layer, while negative charge accumulation is observed near the TaS_2_ layer. This signifies an interfacial charge transfer process, where electrons migrate from the Sc_2_CF_2_ layer to the TaS_2_ layer. The movement of the electrons from the Sc_2_CF_2_ layer to the TaS_2_ layer upon the formation of the TaS_2_/Sc_2_CF_2_ heterostructure describes the formation of the p-type Schottky contact. The p-type doping of the Sc_2_CF_2_ monolayer made its Fermi level shift downwards, so that the band structure of the Sc_2_CF_2_ layer moved upwards relative to the Fermi level of the heterostructure. The calculated amount of transferred charge is approximately 4 × 10^−3^ electrons. Although the charge transfers between the TaS_2_ and Sc_2_CF_2_ layers are small, the electrostatic potential of the TaS_2_/Sc_2_CF_2_ heterostructure is plotted in Fig. 4(c) to analyze the charge distribution across the interface.

A notable charge redistribution is observed near the contact region, indicating the presence of interlayer interactions facilitated by vdW forces. Specifically, positive charge accumulation occurs near the Sc_2_CF_2_ layer, while negative charge accumulation is observed near the TaS_2_ layer, signifying an interfacial charge transfer process where electrons migrate from the Sc_2_CF_2_ layer to the TaS_2_ layer. This electron transfer upon heterostructure formation leads to the establishment of a p-type Schottky contact.

Consequently, the p-type doping effect in the Sc_2_CF_2_ monolayer results in a downward shift of its Fermi level, causing its band structure to move upwards relative to the Fermi level of the heterostructure. The amount of transferred charge is calculated to be approximately 4 × 10^−3^ electrons. Although the charge transfer between the TaS_2_ and Sc_2_CF_2_ layers is relatively small, it induces the formation of an interface dipole, calculated to be 0.50 eV. This relatively small dipole facilitates charge carrier transport, significantly enhancing electron mobility across the heterostructure, which is highly beneficial for electronic applications. Furthermore, the electrostatic potential of the TaS_2_/Sc_2_CF_2_ heterostructure, depicted in Fig. 4(c), provides deeper insights into charge distribution across the interface. The calculated work functions of TaS_2_ and Sc_2_CF_2_ monolayers are 5.34 and 4.85 eV, which are in good agreement with previous reports.^55–57^ The calculated work function of the TaS_2_/Sc_2_CF_2_ heterostructure is intermediate between that of the constituent TaS_2_ and Sc_2_CF_2_ monolayers. The work function of the TaS_2_/Sc_2_CF_2_ heterostructure is found to be 5.02 eV, which is intermediate between those of its constituent layers, thereby confirming the direction of interfacial charge transfer. Additionally, through the electrostatic potential profiles, the tunneling barrier (*Φ*) and tunneling width (*ω*) can be obtained to be 4.75 eV and 1.28 Å, respectively, as depicted in Fig. 4(c). It is obvious that the efficiency of the carrier injection at the interface of the metal–semiconductor TaS_2_/Sc_2_CF_2_ heterostructure can be evaluated *via* the comprehensive factor *C* = *Φ* × *ω*^2^, the tunneling probability *T*_P_ and the specific tunneling resistivity through the Simon model without bias voltage as follows:7
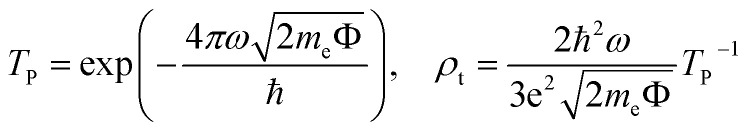


**Fig. 5:**
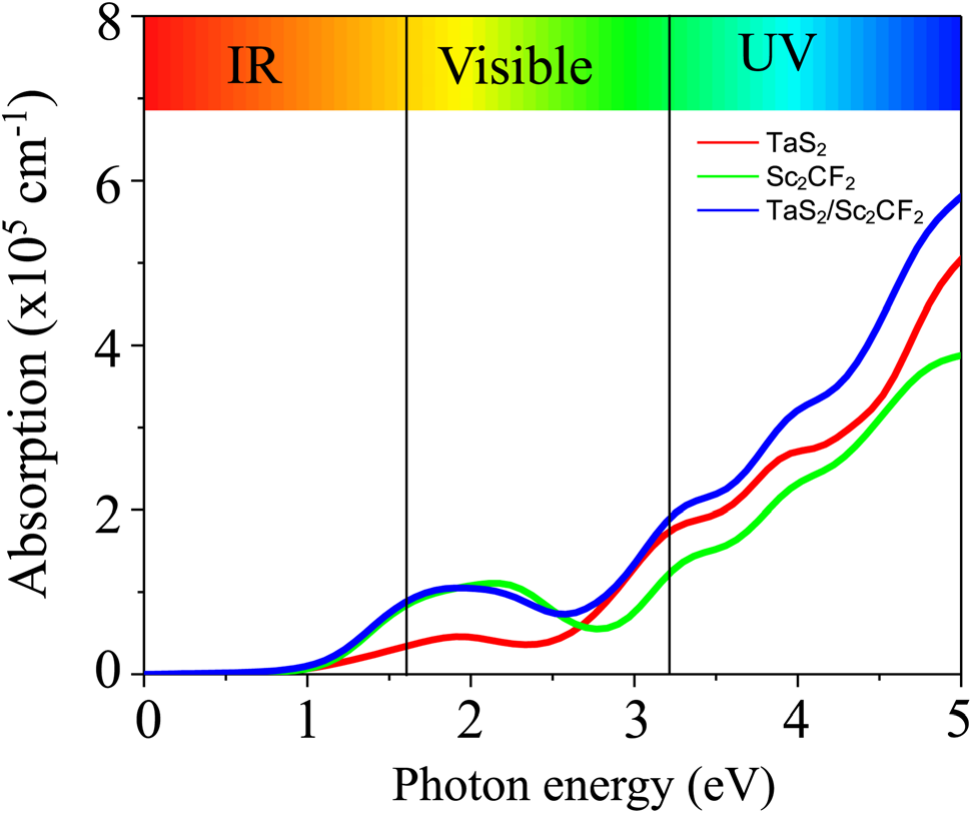
Optical absorption of the TaS_2_/Sc_2_CF_2_ heterostructure for the TS2 stacking configuration.

The obtained *C*, *T*_P_ and *ρ*_t_ of the TaS_2_/Sc_2_CF_2_ heterostructure are to be 7.42 eV.Å2, 6.38% and 1.62 × 10^−10^ Ω cm^2^, respectively. Notably, a lower *C* value corresponds to a higher tunneling probability *T*_P_, leading to enhanced carrier injection efficiency. Moreover, the low *ρ*_t_ value implies that the charge carriers experience minimal obstruction at the interface, supporting efficient carrier injection. This feature is particularly advantageous for the design of high-performance electronic and optoelectronic devices such as transistors and photodetectors. To further explore the optical response, we calculated the absorption spectrum of the TaS_2_/Sc_2_CF_2_Sc_2_CF_2_ heterostructure, as shown in Fig. 5. It is evident that the integration of TaS_2_ and Sc_2_CF_2_ layers leads to a significant enhancement in the absorption coefficient compared with the individual monolayers. Remarkably, the absorption coefficient of the heterostructure reaches up to 6 × 10^5^ cm^−1^, underscoring its strong potential for optoelectronic applications.

**Fig. 6:**
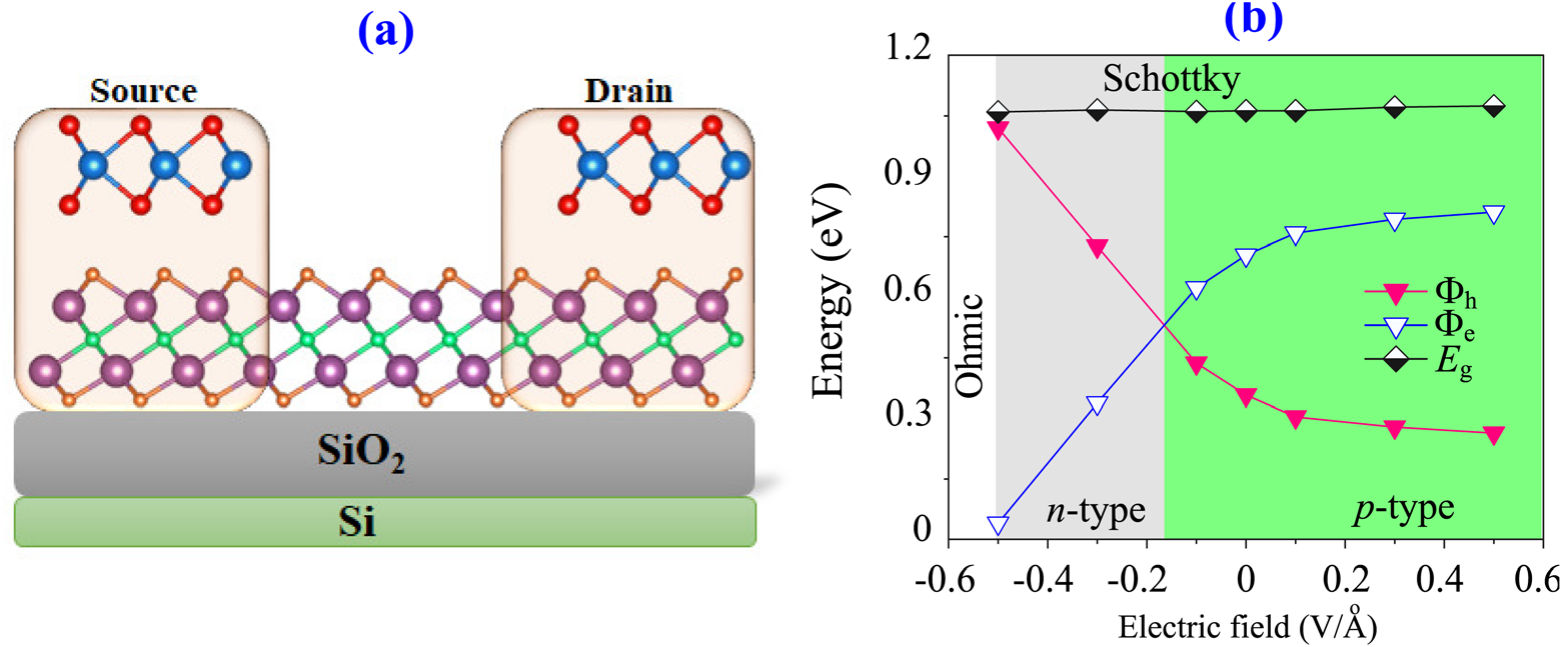
(a) Schematic illustration of the electric field and (b) the tunability of the Schottky barriers and Schottky contact under applied electric fields.

Furthermore, the tunability of the TaS_2_/Sc_2_CF_2_ heterostructure plays a crucial role in optimizing its electronic properties for various applications. Therefore, it is essential to examine how the electronic properties and contact behavior of the TaS_2_/Sc_2_CF_2_ heterostructure respond to an applied electric field. The external electric field was applied perpendicular to the TaS_2_/Sc_2_CF_2_ heterostructure by introducing a sawtooth-like potential. Specifically, the field was oriented along the *z* axis, directed from the TaS_2_ layer toward the Sc_2_CF_2_ layer, as illustrated in Fig. 6(a). It is observed that the electric field induces a transition from Schottky to ohmic contact, as well as a conversion from n-type to p-type Schottky contact, as depicted in Fig. 6(b). For instance, under the application of a positive electric field, the electron barrier *Φ*_e_ increases, while the hole barrier *Φ*_h_ decreases correspondingly. This indicates that the TaS_2_/Sc_2_CF_2_ heterostructure retains its p-type Schottky contact with a reduced hole barrier *Φ*_h_, facilitating enhanced hole injection at the interface. A lower *Φ*_h_ means that the hole injection into the Sc_2_CF_2_ layer is easier, which can significantly improve device performance in applications such as transistors and optoelectronic components, as illustrated in Fig. 6(a). Conversely, under the application of a negative electric field, the electron barrier *Φ*_e_ is narrowed, while the hole barrier *Φ*_h_ is widened correspondingly. As illustrated in Fig. 6(b), when the negative electric field strength drops below −0.2 V Å^−1^, *Φ*_e_ becomes narrower than *Φ*_h_, indicating a transition from a p-type to an n-type Schottky contact. More interestingly, as the negative electric field further decreases beyond −0.5 V Å^−1^, the electron barrier *Φ*_e_ continues to narrow and eventually approaches zero. This signifies a barrier-free charge injection process, facilitating highly efficient electron transport across the heterostructure. This finding indicates a transition toward ohmic contact, where charge carriers can move freely without significant resistance at the interface.

**Fig. 7:**
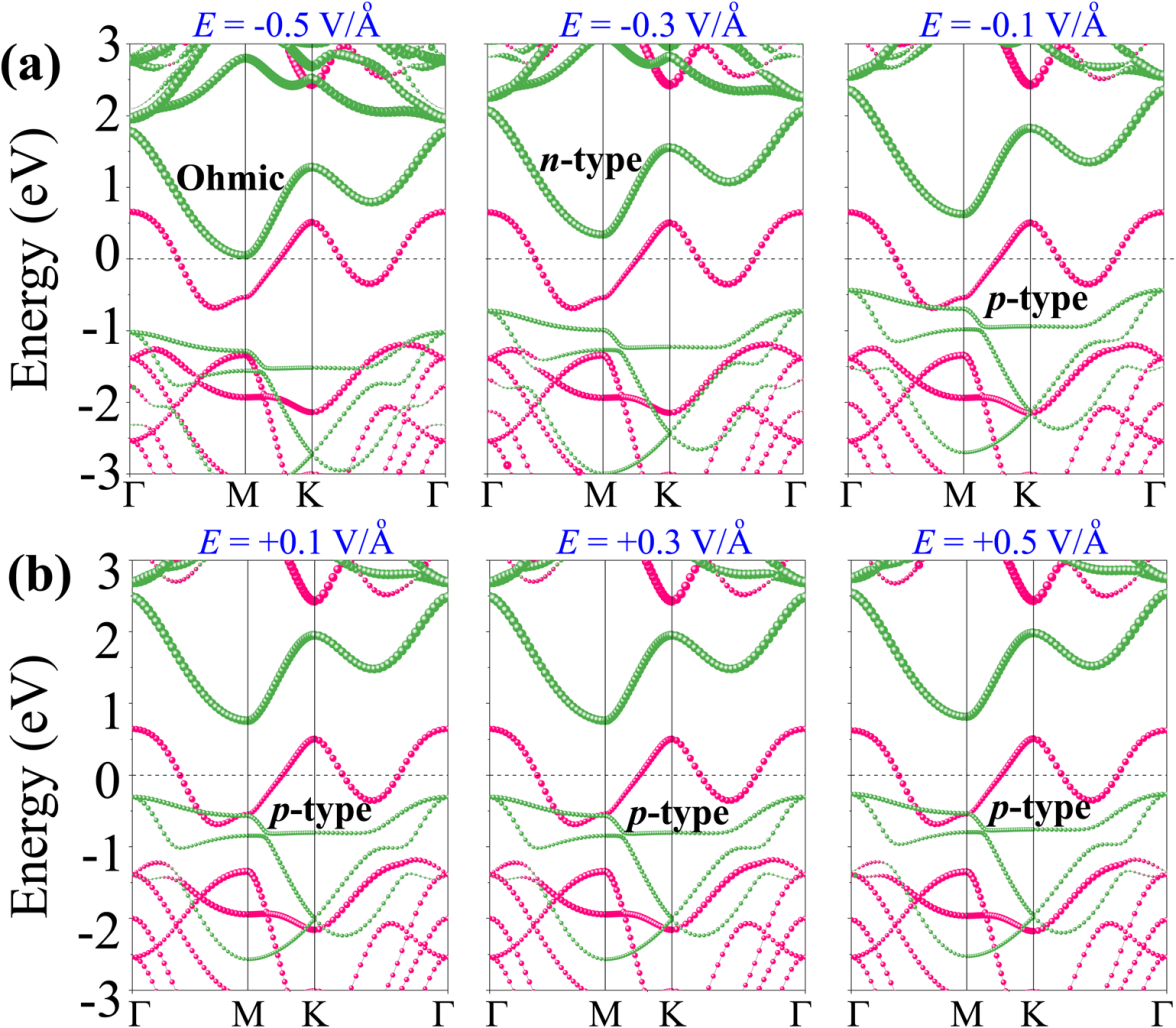
(a) Schematic illustration of the electric field and (b) the tunability of the Schottky barriers and Schottky contact under applied electric fields.

To better understand the physical mechanism underlying the tunability of the TaS_2_/Sc_2_CF_2_ heterostructure, we analyze its projected band structures under varying electric fields, as shown in Fig. 7. As illustrated in Fig. 7(a), the application of a negative electric field induces a downward shift in the band edges of the semiconductor Sc_2_CF_2_ layer toward the Fermi level, effectively reducing the barrier for electron injection. When the negative electric field strength drops below −0.2 V Å^−1^, the CB of the Sc_2_CF_2_ layer moves closer to the Fermi level than the VB, signifying the transition from a p-type to an n-type Schottky contact. At a negative electric field strength below −0.5 V Å^−1^, the CB of the Sc_2_CF_2_ layer crosses the Fermi level, confirming the transition from Schottky to ohmic contact. It is evident that the electric field strongly influences the Sc atoms and the orbital hybridization between C and F atoms, while its effect on the Ta and S atoms is comparatively weak, as illustrated in Fig. S5. This indicates that the energy barrier for electron injection is effectively eliminated, allowing charge carriers to flow freely across the interface without resistance. Fig. 7(b) illustrates the projected band structures of the TaS_2_/Sc_2_CF_2_ heterostructure under the influence of a positive electric field. The band edges of the Sc_2_CF_2_ layer exhibit an upward shift, moving away from the Fermi level. This shift increases the Schottky barrier height for electron injection while reducing the barrier for hole transport, reinforcing the p-type Schottky contact. Furthermore, it should be noted that the amount of transferred charge increases under negative fields, favoring electron injection and ohmic transition, whereas it decreases under positive fields, stabilizing hole-dominated p-type contact. All these findings suggest that the electric fields induce significant modifications in the charge injection mechanism and contact behavior of the TaS_2_/Sc_2_CF_2_ heterostructure, making it a highly tunable system for electronic applications.

## 4 Conclusions

In summary, we employed first-principles calculations to design and investigate the electronic properties and contact performance tunability of the TaS_2_/Sc_2_CF_2_ heterostructure under applying electric fields. Our results demonstrate that the TaS_2_/Sc_2_CF_2_ heterostructure is both energetically favorable and thermally stable, suggesting that it could be synthesized experimentally. The heterostructure is primarily governed by weak vdW interactions, ensuring the preservation of intrinsic properties of its constituent monolayers. Furthermore, the integration of TaS_2_ with Sc_2_CF_2_ enhances the elastic constants and Young modulus, improving mechanical rigidity. More importantly, the TaS_2_/Sc_2_CF_2_ heterostructure forms a Schottky contact with a low Schottky barrier of 0.36 eV, which can be actively tuned *via* an applied electric field. Under a negative electric field, the system undergoes a transition from Schottky to ohmic contact, alongside a conversion from n-type to p-type Schottky contact. This tunability facilitates barrier-free charge injection, positioning the TaS_2_/Sc_2_CF_2_ heterostructure as a promising candidate for next-generation electronic and optoelectronic applications, such as transistors and photodetectors.

## Conflicts of interest

There are no conflicts to declare.

## Supplementary Material

RA-015-D5RA05385D-s001

## Data Availability

The data that support the findings of this study are available from the corresponding author upon reasonable request. Supplementary information is available. See DOI: https://doi.org/10.1039/d5ra05385d.
