# Supplementary material for: Two-dimensional TaS2 as a contact material for MXene Sc2CF2 semiconductors: a first-principles study
Source: RSC Adv. 2025 Oct 20;15(47):39696–704. doi: 10.1039/d5ra05385d (PMC12536630; doi:10.1039/d5ra05385d)
Supplement: RA-015-D5RA05385D-s001 [file RA-015-D5RA05385D-s001.pdf]

Supplementary Information (SI) for RSC Advances.
This journal is © The Royal Society of Chemistry 2025

Supporting Information (SI) for

Two-Dimensional TaS_2 as a Contact Material for MXene Sc_2CF_2 Semiconductors: A First-Principles Study

Tuan V. Vu[1,2], Phan T. T. Huyen[3], Nguyen N. Hieu[4,5,†], Huynh V. Phuc[6,†], and Chuong V. Nguyen[7]

[1]*Laboratory for Computational Physics, Institute for Computational Science and Artificial Intelligence, Van Lang University, Ho Chi Minh City, Vietnam. E-mail: tuan.vu@vlu.edu.vn*
[2]*Faculty of Mechanical, Electrical, and Computer Engineering, Van Lang School of Technology, Van Lang University, Ho Chi Minh City, Vietnam*
[3]*Hue FPT School, Hue, Vietnam*
[4]*Institute of Research and Development, Duy Tan University, Da Nang 550000, Vietnam. E-mail: hieunn@duytan.edu.vn*
[5]*Faculty of Natural Sciences, Duy Tan University, Da Nang 550000, Viet Nam*
[6]*Division of Physics, School of Education, Dong Thap University, Cao Lanh 870000, Vietnam. Email: hvphuc@dthu.edu.vn*
[7]*Department of Materials Science and Engineering, Le Quy Don Technical University, Hanoi 100000, Vietnam*
†*To whom correspondence should be addressed*

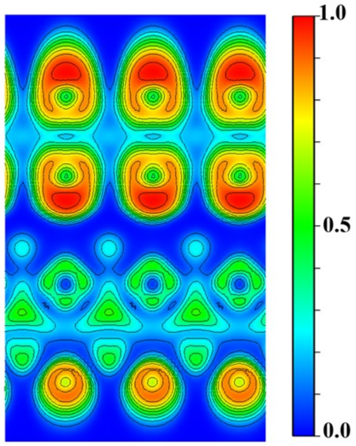

Fig. S1. Electron localization function (ELF) map of the TaS2/Sc2CF2 heterostructure for the most energetically favorable stacking configuration TS2. A value of 1.00 corresponds to fully localized electrons, 0.50 to fully delocalized electrons, and 0.00 to regions of very low charge density.

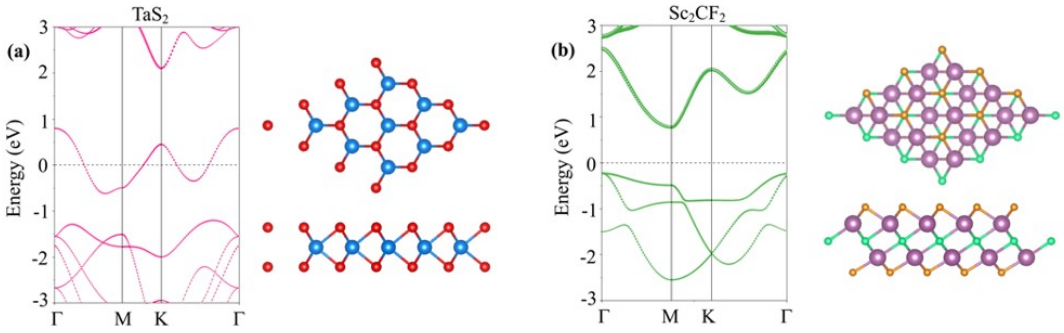

Fig. S2. Band structures and atomic structures of (a) TaS2 and (b) Sc2CF2 monolayers. Red and blue balls represent the S and Ta atoms, respectively. Yellow, purple and green balls stand for the F, Sc and C atoms, respectively.

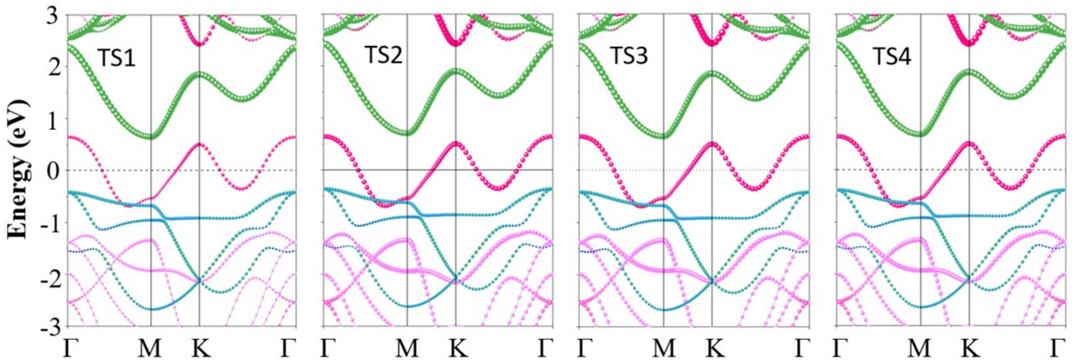

Fig. S3. Element-projected band structures of the TaS2/Sc2CF2 heterostructure for different stacking configurations. The weighted contributions from each element are highlighted: Ta (pink), S (purple), Sc (green), C (cyan), and F (blue).

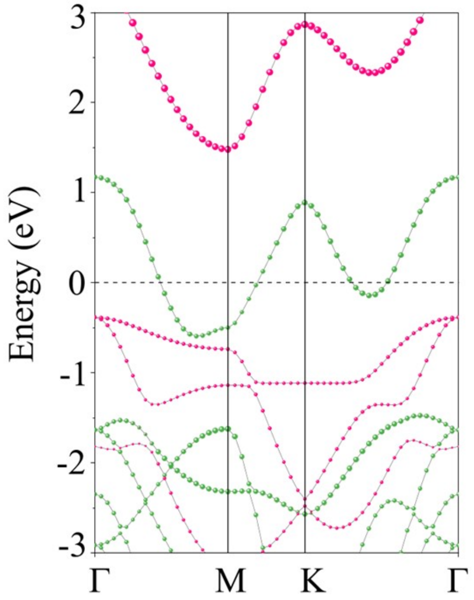

Fig. S4. HSE projected band structures of TaS2/Sc2CF2 heterostructure for the stacking TS2 configuration.

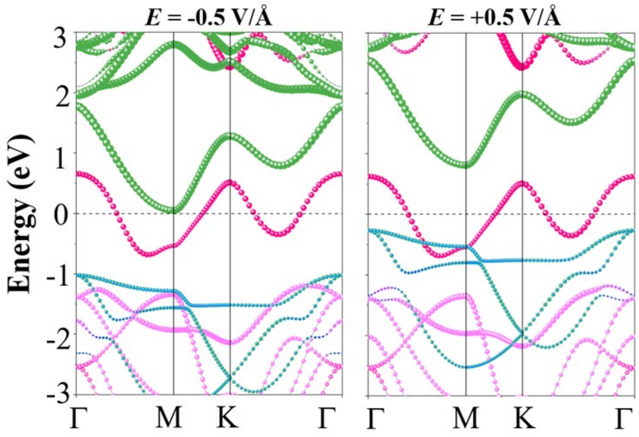

Fig. S5. Element-projected band structures of the TaS2/Sc2CF2 heterostructure in the TS2 stacking configuration under different external electric fields. The weighted contributions from each element are highlighted: Ta (pink), S (purple), Sc (green), C (cyan), and F (blue).
